# Ultimate mechanical properties of enstatite

**DOI:** 10.1007/s00269-022-01206-5

**Published:** 2022-07-11

**Authors:** Karine Gouriet, Pascal Roussel, Philippe Carrez, Patrick Cordier

**Affiliations:** 1 University Lille, CNRS, INRAE, Centrale Lille, UMR 8207-UMET-Unité Matériaux et Transformations, 59000 Lille, France; 2 University Lille, CNRS, Centrale Lille, University Artois, UMR 8181, Unité de Catalyse et Chimie du Solide, 59000 Lille, France; 3 Institut Universitaire de France, 1 rue Descartes, 75005 Paris, France

**Keywords:** Orthoenstatite, Ultimate mechanical properties, Ideal tensile strength, Ideal shear strength

## Abstract

The ultimate mechanical properties of MgSiO_3_ orthoenstatite (OEN), as characterized here by the ideal strengths, have been calculated under tensile and shear loadings using first-principles calculations. Both ideal tensile strength (ITS) and shear strength (ISS) are computed by applying homogeneous strain increments along high-symmetry directions ([100], [010], and [001]) and low index shear planes ((100), (010), and (001)) of the orthorhombic lattice. We show that the ultimate mechanical properties of OEN are highly anisotropic during tensile loading, with ITS ranging from 4.5 GPa along [001] to 8.7 GPa along [100], and quite isotropic during the shear loading with ISS ranging from 7.4 to 8.9 GPa. During tensile test along [100] and [001], a modified structure close to OEN has been found. This modified structure is more stable than OEN under stress (or strain). We have characterized its elastic and ultimate properties under tensile loading. With ITS ranging from 7.6 GPa along [010] to 25.6 GPa along [001], this modified structure appears to be very anisotropic with exceptional strength along [001].

**Supplementary Information:**

The online version contains supplementary material available at 10.1007/s00269-022-01206-5.

## Introduction

The mechanical properties of crystals are usually controlled by defects. In the brittle field microcracks, pores, inclusions control toughness. In the ductile field, flow results from the motion of vacancies, dislocations, twins, disclinations, disconnections, etc. However, in some circumstances, these mechanisms cannot be activated and the mechanical properties of solids are ultimately controlled by the resistance of bonds. This is the case when plastic deformation mechanisms are inhibited by temperature being too low, or by kinetics reasons at high strain rates. The first approach to this problem was formulated by Frenkel (1926) who predicts the ideal shear stress to be on the order of $G/2\pi$ ($G$ is the shear modulus). Ideal (shear or tensile) stresses are difficult to approach experimentally, but they are now easily accessible using pseudopotential total energy calculation based on the density functional theory (DFT) (Sob et al. 1997; Roundy et al. 1999; Ogata et al. 2004). In simple solids, the correlation between ultimate and elastic properties is quite good, thus validating Frenkel's theoretical approach (Ogata et al. 2004). However, with more complex structures, deviations may occur. Jiang and Srinivasan (2013) have shown that in Fe_3_C new bonds are formed during deformation leading to a strong strain-stiffening of the structure. Only calculations at the atomic scale can predict the ultimate properties of these solids.

(Mg, Fe)SiO_3_ is one of the most abundant planetary materials, present in planetary mantles, but also in interplanetary dust particles (Rietmeijer, 1998) and chondritic meteorites (Brearley and Jones 1998). During the formation of the solar system, the minerals that make up these objects were subjected to multiple collisions that could lead to the destabilization of the crystalline structure. Hernandez et al. (2020) have recently shown disordering in shock-compressed enstatite. However, contrary to olivine (Gouriet et al. 2019; Misawa and Shimoyo 2020), the ultimate properties and mechanical stability of enstatite have not yet been documented.

In this study, we investigate the elastic properties and mechanical stability of iron-free MgSiO_3_ orthoenstatite. From first-principles calculations, the ideal tensile strengths (ITS) and ideal shear strengths (ISS) are computed along high-symmetry directions [100], [010], and [001] and for homogeneous shear on (100), (010), and (001) planes (here given with respect to the *a* ~ 18.4 Å, *b* ~ 8.9 Å, *c* ~ 5.2 Å cell parameters’ setting of orthoenstatite).

## Methods

We performed the derivation of the anisotropic ideal strengths of OEN using the ADAIS free software, written by Zhang et al. (2019). The ADAIS software allows us to implement a homogenous deformation to standard first-principles VASP calculations (Kress and Frurthmüller 1996). Following the extended work of Li et al. (2014) on enstatite polymorphs, all calculations are based on DFT using a plane-wave basis set and the projector augmented wave method (PAW) (Perdew and Wang 1992). The Perdew–Wang (PW91) gradient-corrected functional (GGA) (Wang and Perdew 1991) is employed to take into account the exchange–correlation energy. To ensure suitable atomic force convergence, we used a kinetic energy cutoff of 520 eV for the expansion of the plane-wave basis set. In this work, we use a single grid of 2 × 4 × 6 for the k-points sampling, according to a Monkhorst and Pack scheme (Monkhorst and Pack 1976) corresponding to 18 k-points per enstatite (MgSiO_3_) unit cell.

**Fig. 1:**
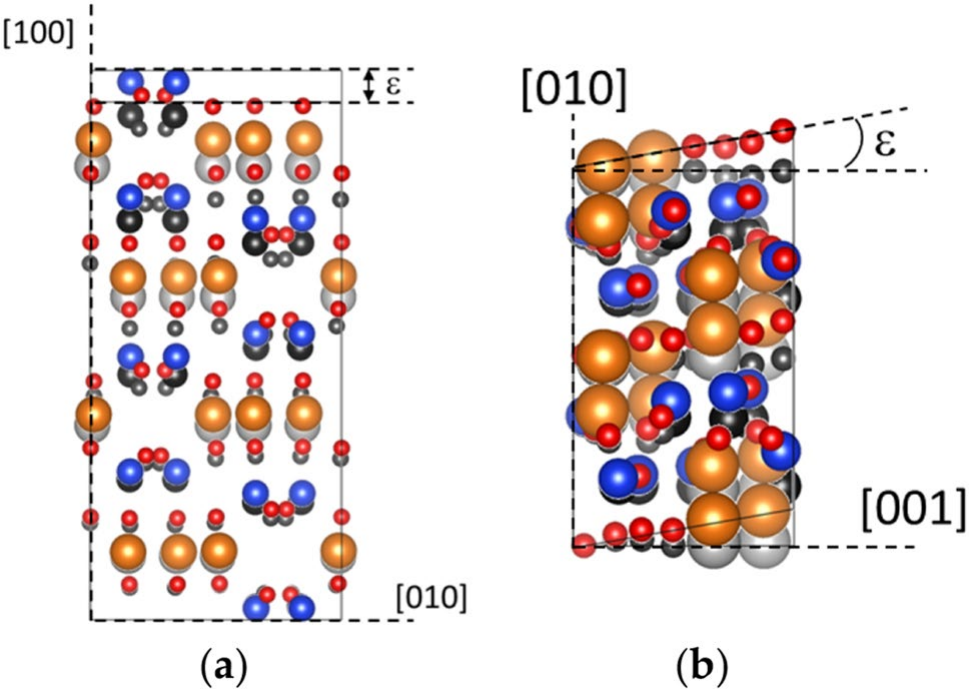
Illustration of the various loading conditions applied in this study: (**a**) tensile deformation along [001] where *ε*_*zz*_ = *ε*; (**b**) simple shear [001](100) where *ε*_*xz*_ = *ε*/2 and *ε*_*zx*_ = 0. The image in gray is the structure at *ε* = 0, in color at a given *ε*. Mg is in orange, Si in blue, and O in red

Starting from a fully optimized unit cell of MgSiO_3_ OEN, we performed tensile and shear tests by applying a homogenous strain incrementally by step of 0.5%. In practice, crystal atomic layers are moved along the tensile or shear directions (Fig. 1). At each deformation stage, the cell shape and the atomic positions are relaxed, until all the components of the stress tensor are brought to zero, except for the one corresponding to the applied stress condition.

## Results and discussion

### Ground properties

Prior to mechanical testing, the unit cell of OEN (containing 80 atoms) is optimized. The ground-state properties, equilibrium lattice parameters, and elastic constants are given in Tables 1 and 2, respectively. Lattice parameters are found in agreement with experimental values (Jackson et al. 2007; Weidner and Vaughan 1982). The elastic constants calculated here for OEN are consistent with those published by Li et al. (2014). Nevertheless, one may notice the well-documented GGA approximation tendency to underestimate the elastic constants with respect to their experimental counterparts (Table 2). As discussed later, it is worth to recall that one of the particularities of the OEN structure consists in alternating SiO_4_ tetrahedra chains connected to octahedral Mg1 and Mg2 sites layers along the c-axis and in (100) layers (Periotto et al. 2012) (see suppl. Figure 1). Moreover, one notes that the octahedron on Mg2 site is larger and distorted, compared to the Mg1 octahedron (see suppl. Figure 1b).

**Table 1:** Orthoenstatite

	*a* (Å)	*b* (Å)	*c* (Å)	*V* (Å^3^)
This study	18.399	8.919	5.235	859.05
Calculated GGA (Li et al. 2014)	18.417	8.9345	5.2448	863.038
Calculated LDA (Li et al. 2014)	18.072	8.6889	5.1194	803.897
Experimental (Periotto et al. 2012)	18.2104	8.820	5.1767	821.4
Experimental (Duffy and Vaughan, 1988)	18.204	8.814	5.176	830.4

Crystallographic data for OEN at ambient conditions (RT and 0 GPa) compared with values calculated at 0 K and 0 GPa

**Table 2:** Orthoenstatite

	*C*_11_	*C*_22_	*C*_33_	*C*_44_	*C*_55_	*C*_66_	*C*_12_	*C*_13_	*C*_23_
Jackson et al. (2007)	225	178	214	78	76	82	72	54	53
Weidner and Vaughan (1982)	236	173	216	84	79	80	74	57	50
Calculated GGA (Li et al. 2014)	200	146	147	76	63	70	59	38	29
Calculated LDA (Li et al. 2014)	251	195	241	89	80	86	90	77	71
Orthoenstatite GGA (this study)	205	147	116	70	65	74	60	37	27

Elastic constants (in GPa) for OEN at 0 GPa compared with previous works

### Ideal tensile test calculations

**Fig. 2:**
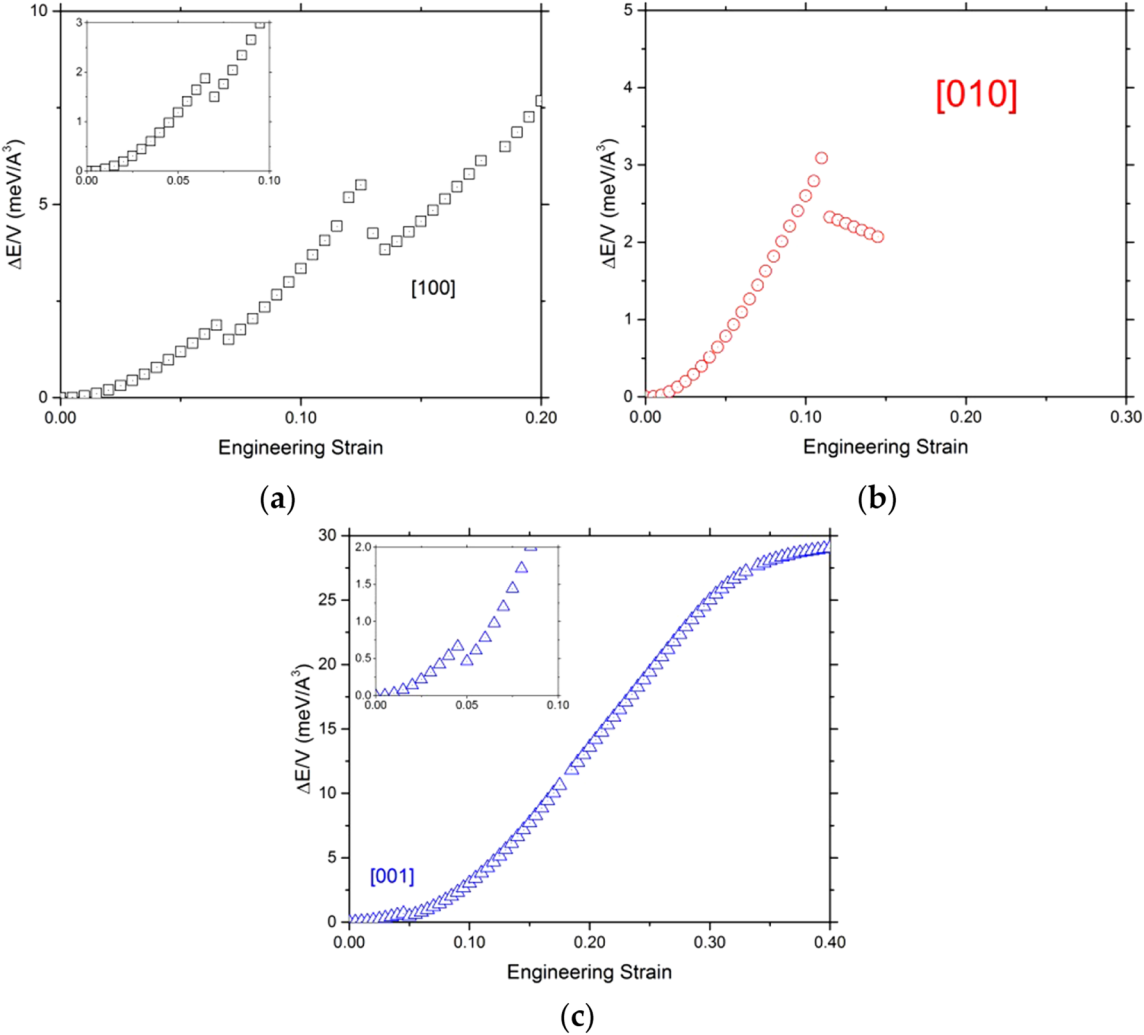
Evolution of the total energy as a function of the engineering strain. The tensile directions are (**a**) [100] (black empty square), (**b**) [010] (red empty circle), and (**c**) [001] (blue empty triangle). Insert plots shown a zoom of the beginning of [001] and [001] curves

**Fig. 3:**
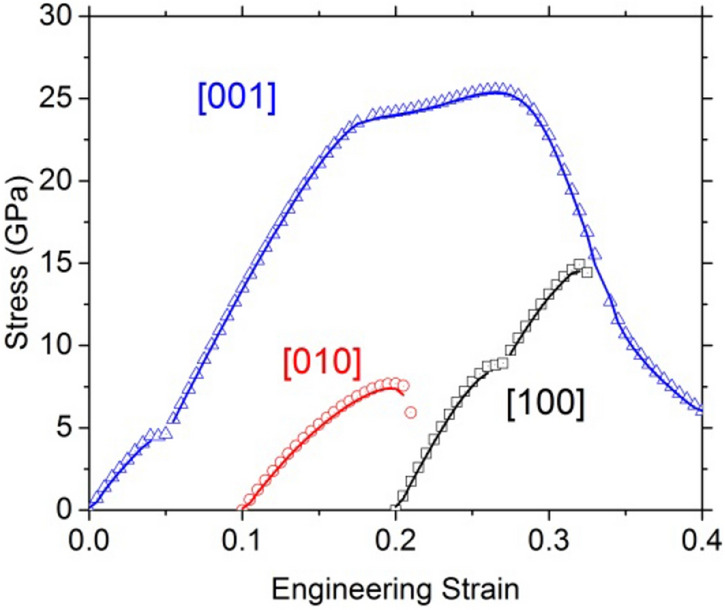
Evolution of the stress as a function of the engineering strain. The tensile directions are [100] (black empty square), [001] (blue empty triangle), and [010] (red empty circle). The instability occurs at the first incident on the curves

Tensile tests are performed along the [100], [010], and [001] directions. The evolution of the total strain energy as a function of the engineering strain is shown in Fig. 2. First, we observe for all curves a parabolic evolution, which corresponds to the storage of the elastic energy. These parabolic parts correspond to the linear portion on the stress–strain curves shown in Fig. 3.

For both [100] and [001] tensile tests, we observe a discontinuity in the energy curves near 5% strain. However, for the test along [001], after this first discontinuity, the energy curve increases again until it bends at energy values much higher than those reached in the other two directions. The evolution of the energy for the [100] solicitation presents the same feature followed by a monotonous increase until a second energy drop, but of a larger amplitude. The test along [010] shows only one energy drop at 12%, after which the energy decreases continuously with strain, suggesting that above 12% strain, the OEN structure is no longer stable.

The Cauchy stress can be obtained from the derivation of the energy (solid line in Fig. 3). It agrees with the stress state (according to the Hellman–Feynman theorem) of the strained volume (symbols in Fig. 3). The maximums of the stress–strain curves correspond to an inflexion point of the energy–strain curves. Unsurprisingly, the stress–strain curves (Fig. 3) highlight the discontinuities observed on the energy curves. If generally, the maximum of the stress–strain curves corresponds to the ideal strength, it is necessary to understand the origin of these incidents before discussing the observed mechanical properties. To do this, we study the evolution of the structure during deformation, taking the Mg–O bonds as an indicator.

### Evolution of bonding and structural changes

**Fig. 4:**
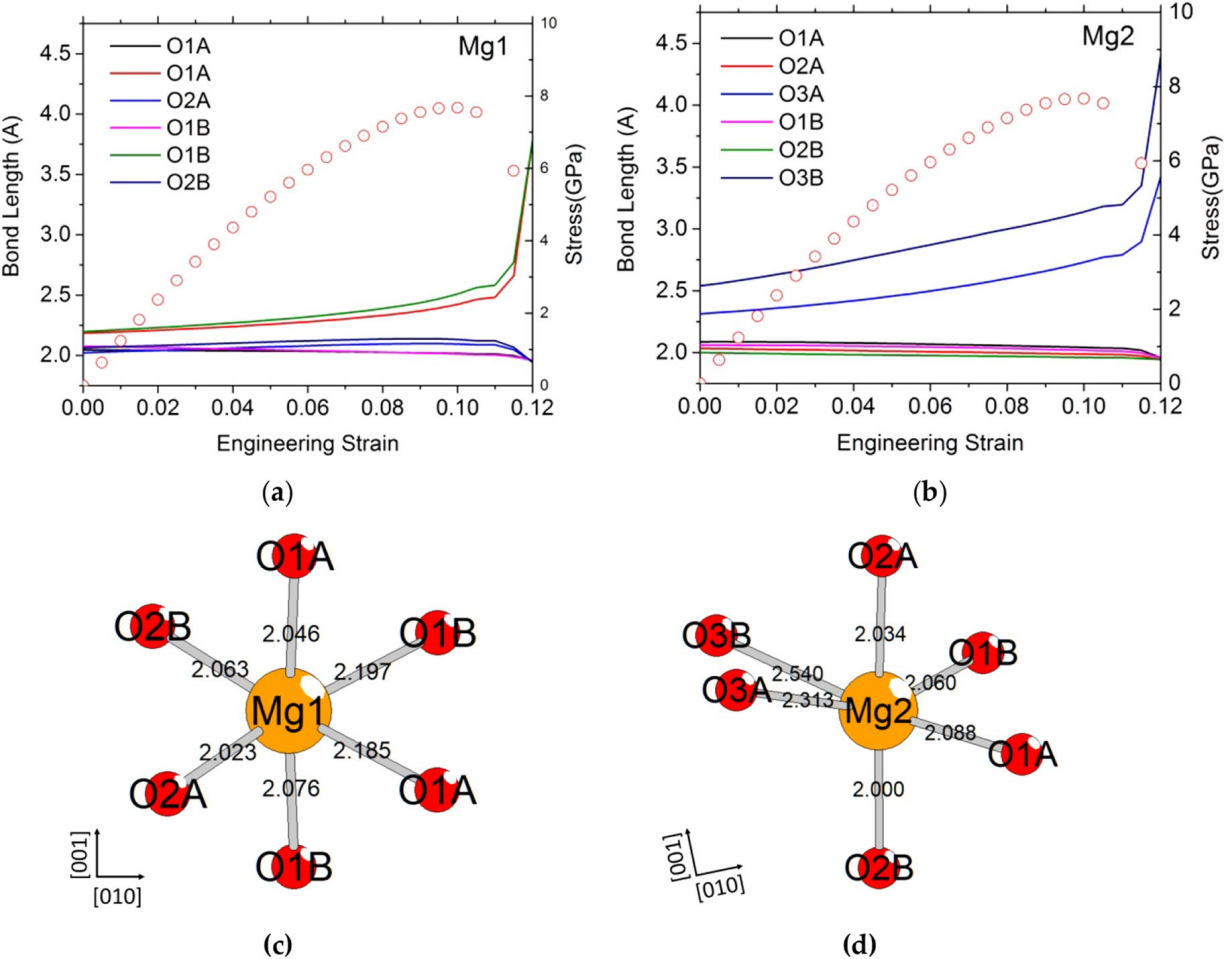
Typical Mg–O-bond length evolution as a function of strain for tensile deformation of the enstatite along [010] for Mg1 (**a**) and Mg2 (**b**) sites. To help the reader, we plot also the stress–strain curves. (**c**) Mg1 (**d**) Mg2 octahedra showing the oxygen label and the initial Mg–O-bond lengths

The tensile test along [010] exhibits the simplest behavior. During [010] tensile loading, only the four longest Mg–O bonds are affected: two for the Mg1 site (Fig. 4a/c) and two for the Mg2 site (Fig. 4b/d), see Suppl. Figure 1 for the definition of each site in the enstatite structure. The bond length evolution is small until the maximum value of the stress is reached, where the four Mg–O-bond lengths diverge, as the instability of the structure appears. All equivalent Mg1 and Mg2 exhibit the same behavior. The Mg2 site corresponds to the most distorted octahedron. Then, the longest Mg–O bond (here the bond between Mg2 and O3B) breaks first. Pulling enstatite along [010] leads to a simple behavior with an elastic loading until mechanical instability by Mg–O-bond breaking. The corresponding ITS is 7.6 GPa at 11% strain.

**Fig. 5:**
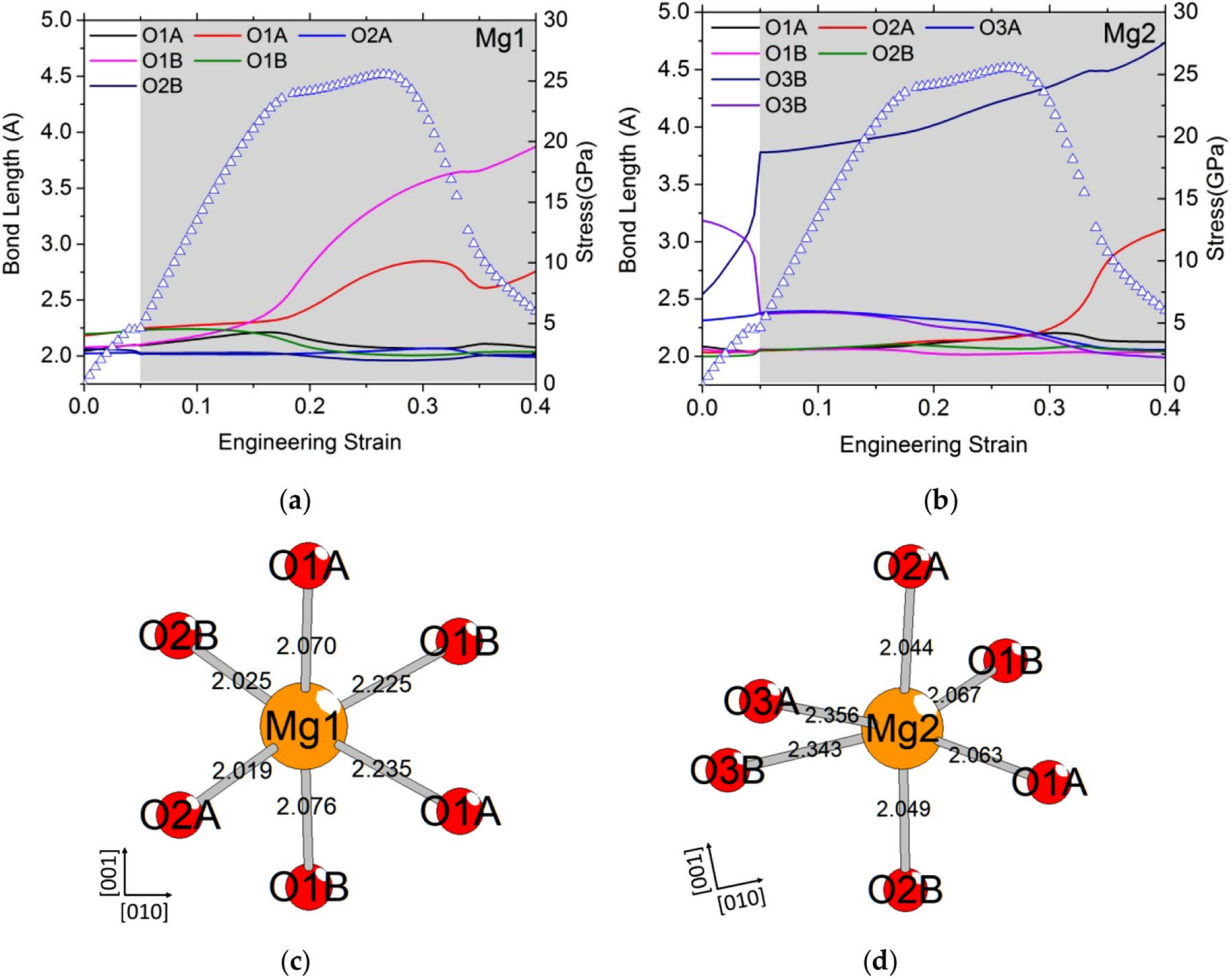
Typical Mg–O-bond length evolution as a function of strain for tensile deformation along [001] for Mg1 (**a**) and Mg2 (**b**) sites. To help the reader, we plot also the stress–strain curves. The gray zone corresponds to the modified structure. (**c**) Mg1 and (**d**) Mg2 octahedra showing the oxygen label and the initial Mg–O-bond lengths, for the relaxed modified structure at 0% of strain

**Fig. 6:**
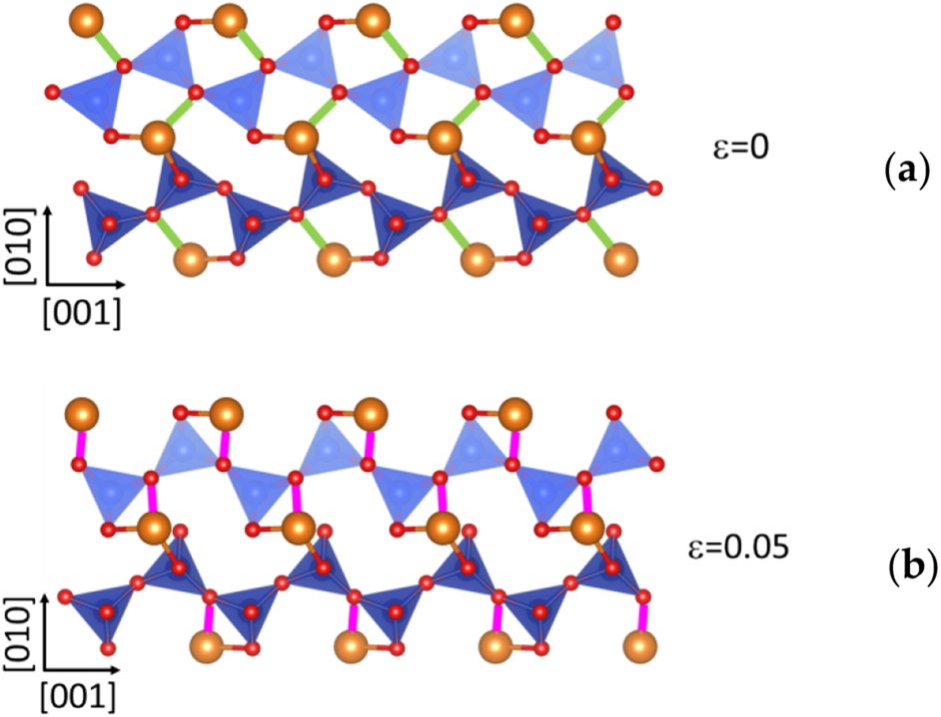
Chain B of tetrahedral sites: (**a**) without deformation, enstatite structure (**b**) modified structure at 6% of strain for tensile deformation along [001]. The Mg–O bonds in green correspond to the lost bond after 6% of deformation. The Mg–O bonds in purple correspond to the new Mg–O bond. The Mg are in orange, O in red, and Si in blue

For the tensile test along the [001] direction, the behavior of Mg in site 2 sheds some light on the event observed at 5% strain where we observe the complete loss of one bond simultaneously with another being re-built (Fig. 5b). Here, bonds of two O3 type oxygen atoms in the B layer are exchanged due to the tilting of the tetrahedra (Figs. 5d, 6). The deformation is accommodated by the elongation of the chain resulting from this tilting (Fig. 6b). This modified structure becomes progressively unstable as, at 16% strain, two Mg1–O-bond lengths increase rapidly and one breaks after 20% strain (Fig. 5a). Then, at 26.5%, an Mg1 loses the second bond, and finally, at 32.5%, an octahedron of the Mg2 site loses one oxygen bond.

**Fig. 7:**
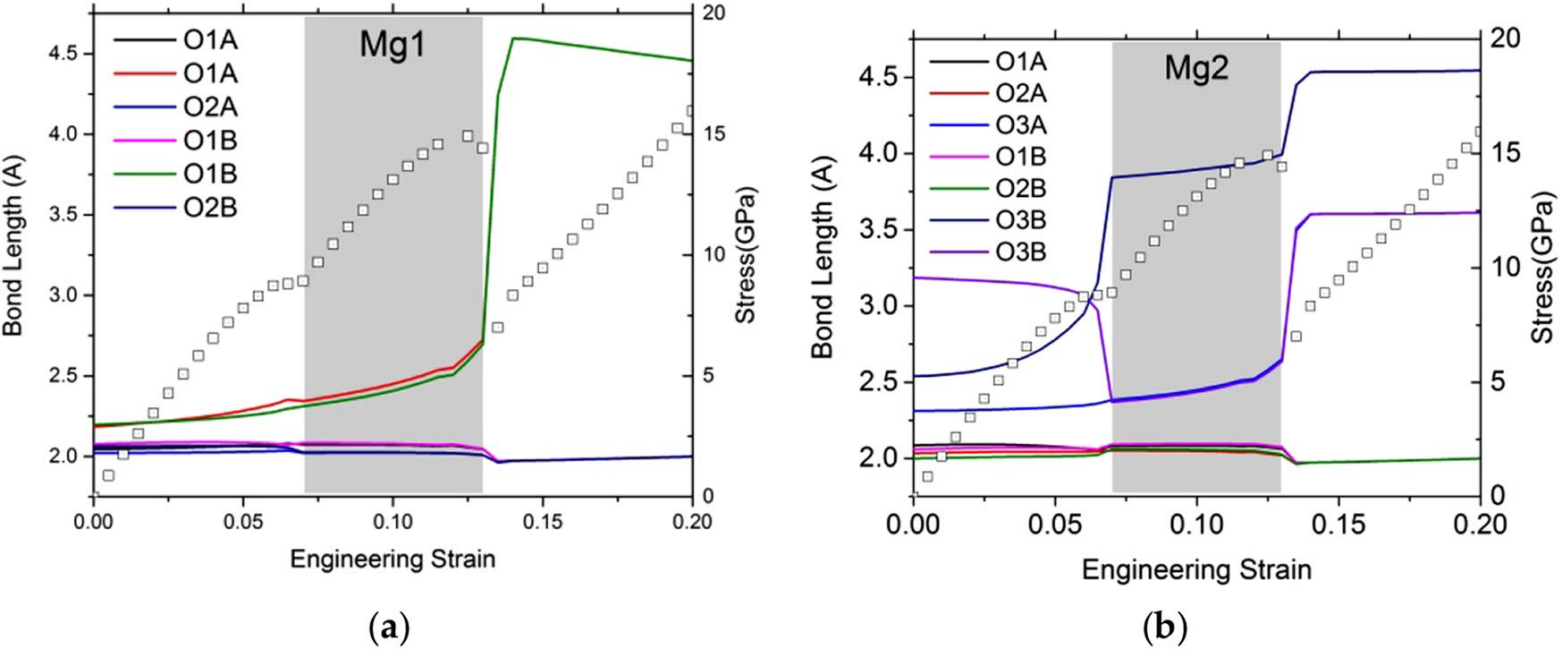
Typical Mg–O-bond length evolution as a function of strain for tensile deformation along [100] for Mg1 (**a**) and Mg2 (**b**) sites. To help the reader, we plot also the stress–strain curves. The gray zone corresponds to the modified stress–strain curve

The analysis of the tensile test along the [100] direction leads to similar conclusions, with after 8% of strain an exchange of oxygen for the Mg in the site 2 (Fig. 7b) and the formation of the same modified structure as the one described in Fig. 6. After this bond switch, the Mg–O bonds are maintained until the instability occurs at 12%, where four Mg–O bonds are broken (Fig. 7). Here, the distorted octahedron plays an important role in the accommodation of the deformation. After 12%, the modified enstatite structure is finally destabilized.

**Fig. 8:**
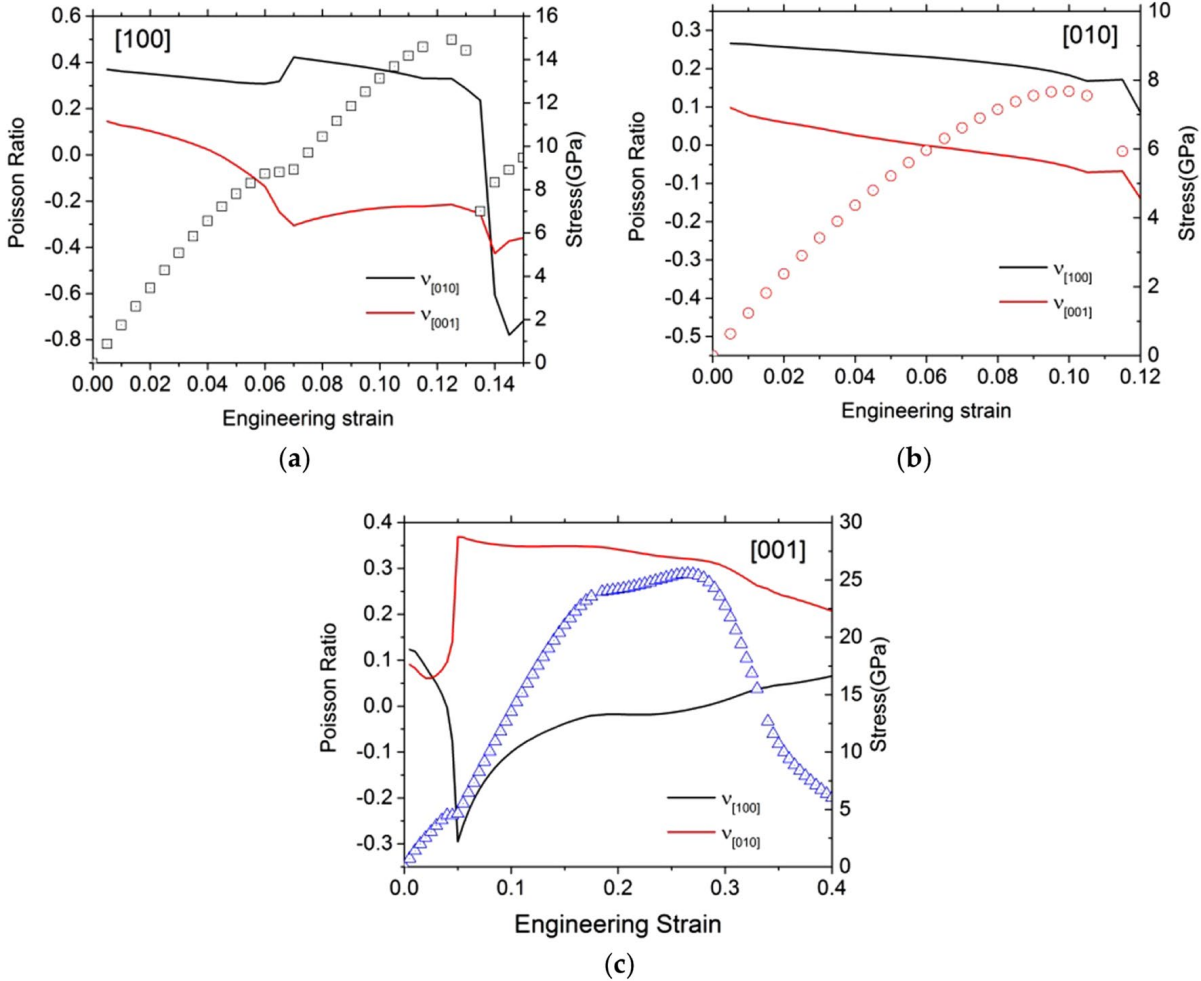
Apparent Poisson ratio (line) as a function of the engineering strain calculated using a unit cell of forsterite under tensile deformation for (**a**) along [100], (**b**) along [010], and (**c**) along [001]. To help the reader, we plot also the stress–strain curves (symbols)

In Fig. 8, we plot the ratio of the longitudinal over the transversal strains and their evolutions with the engineering strain. We see that this parameter, which is an apparent Poisson ratio, although it is used here beyond its natural field of application which is the linear elasticity, represents a good indicator of structural changes. For example, a clear jump is observed with the exchange of O bonds for Mg2 (Fig. 8a, c) with a marked evolution preceding the structure change (especially for the tensile test along [001]). Comparatively, the evolution is less pronounced at the approach of instability. We also observe several occurrences of the apparent Poisson ratio becoming negative, which indicates that enstatite becomes rapidly auxetic under anisotropic strain.

### Modified enstatite: structure and ultimate mechanical properties

We have further investigated the modified structure obtained at 5–6% strain during tension along [001] and [100]. Once formed, it can be kept when stress is released, as a metastable structure. Indeed, as expected, without applied strain, its energy is larger (∆*E* = 0.121 eV/unit cell) than the one of OEN which is the stable polymorph of MgSiO_3_ under these conditions. However, above 5% tensile strain along [001] and 8% strain along [100], this modified structure is more stable as shown by the energy drop (Fig. 2).

Even if, as usual, the modified structure has been relaxed without symmetry constraint in the cell, an analysis of the "missed symmetry elements" of the resulting relaxation was undertaken using the ADDSYM tool, as implemented in the Platon software (Spek 2020), a significantly extended/modified implementation of the MISSYM algorithm (Le Page 1987) for the detection of possibly MISsed ADDitional SYMmetry in a given coordinate set. It turns out that this modified structure also adopts the *Pbca* space group of OEN, with relaxed lattice parameters: *a* = 18.727 Å, *b* = 8.837 Å and *c* = 5.363 Å. The modified structure retains the main particularity of the OEN structure, consisting in alternating SiO_4_ tetrahedron chains and octahedral Mg1 and Mg2 sites’ layers along the *c*-axis and in (100) layers. However, the octahedron on Mg2 site of the modified structure is less distorted than the one of OEN, since, due to the exchange of two O3B oxygens, the bond lengths of both new O3B and O3A are equivalent and close to 2.35 (Figs. 4d and 5d and Table 3), compared to 2.54 and 2.31, respectively. For the octahedron on Mg1 site, almost no change is observed, except the lengths of the two longest bonds that increase by 5% after formation of the modified structure (Figs. 4c and 5c and Table 3). Concerning the silicon surrounding, if on one hand, the tetrahedral chain A does not suffer from the deformation (the characteristic tetrahedron angle (O3A–O3A–O3A) (Cameron and Papike 1981) changes only by 2.5°); on the other hand, a strong modification is observed for the tetrahedron chain B, due to the lengthening of the chain. The O3B–O3B–O3B angle goes from 139.9° to 158.2° (Supp. Figure 3). In summary, after deformation, both tetrahedron chains tend towards the same configuration, with characteristic angles for the tetrahedral chain close to 160°. Finally, calculated powder X-ray diffractions using Cu *K*α_1_ radiation have been calculated for OEN and the modified structure, and are given in Supp. Figure 4. It is interesting to note a strong difference between the two diffractogramms, giving hope to strong changes in the diffraction pattern, and hence an easy signature to evidence a transition, if an experimental strain is applied along this direction. The modified structure is different from the high-pressure structures found by Jahn (2008) with first principle calculations, called HP-OEN1 in Jahn (2008) or *Pbca*-II in Li et al. (2014). Furthermore, at 0 GPa, the difference of internal energy per unit cell between the HP-OEN1 structure found in Jahn (2008) and OEN is below 0.1 eV/unit cell, whereas, here, we computed for the modified structure an increase of the internal energy of 0.121 eV/unit cell. If the structural modifications appear in layer B in the modified structure, only the layer A is affected in HP-OEN1 (Jahn 2008).

**Table 3:** Bond length (in Å) of Mg2–O and Mg1–O, and the O3–O3–O3 (°) angles for orthoenstatite (OEN) and the modified structure (MS) at 0 GPa, compared with experimental data for the OEN

	OEN	Periotto et al. (2012)	MS
Mg1-O1A	2.046	2.020	2.070
Mg1-O1A	**2.185**	**2.146**	**2.235**
Mg1-O1B	**2.197**	**2.162**	**2.235**
Mg1-O1B	2.076	2.064	2.076
Mg1-O2A	2.023	2.006	2.019
Mg1-O2B	2.063	2.049	2.025
Mg2-O1A	2.088	2.096	2.063
Mg2-O1B	2.060	2.057	2.067
Mg2-O2A	2.034	2.032	2.044
Mg2-O2B	2.000	1.986	2.049
Mg2-O3A	**2.313**	**2.297**	**2.356**
Mg2-O3B	**2.540**	**2.450**	**2.343**
O3A–O3A–O3A	157.6	159.3	160.1
O3B–O3B–O3B	139.9	139.4	158.2

The longest bonds are in bold

**Fig. 9:**
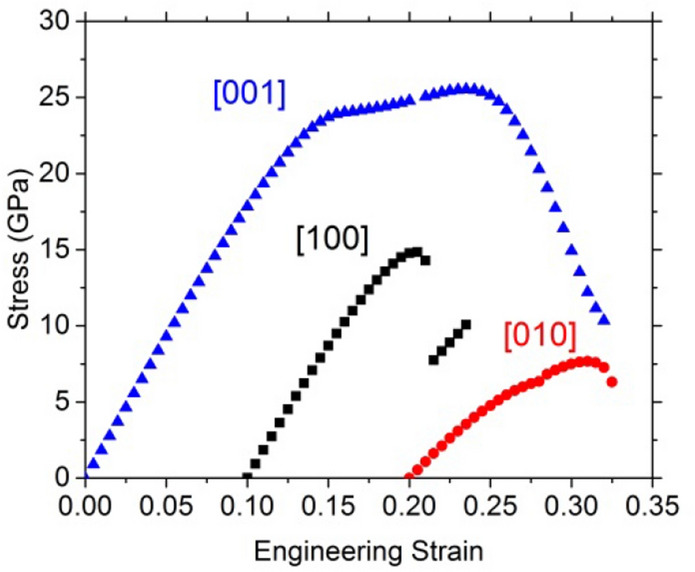
Modified structure. Stress as a function of the engineering strain calculated using a unit cell of modified enstatite for the tensile tests along [100] direction in black square, [010] direction in red circle, and [001] blue triangles

Since we can preserve this modified structure in a metastable state, and relax it at 0% strain, we can study its elastic properties. The nine elastic constants computed for the modified structure are given in Table 4. It is worth noticing that all *C*_*ij*_ are positive and meets the criteria for mechanical stability of orthorhombic structure, confirming, therefore, the metastability of the MS phase. The results show a larger value of C_33_ than OEN and more isotropic shear moduli (Table 4). We ran also tensile tests along the three directions with this modified structure after relaxation (Fig. 9) which reproduce very well the behavior obtained previously after 4–5% strain (Supp. Figure 2), demonstrating that this portion of the curves after the incidents describes the behavior of this modified structure and not the one of OEN.

**Table 4:** Modified structure

	*C*_11_	*C*_22_	*C*_33_	*C*_44_	*C*_55_	*C*_66_	*C*_12_	*C*_13_	*C*_23_
Modified orthoenstatite	220	148	211	51	56	71	69	53	60

Elastic constants (in GPa) for relaxed modified structure at 0 K and 0 GPa at 0% of strain

The modified enstatite is very anisotropic with respect to the ideal tensile test investigated. For the [010] tensile test, a small leap appears at 8%, without incident on the structure. The ITS along [100] and [001] are 14.8 and 25.6 GPa, respectively. Along [001], we observe a plateau after 15% strain corresponding to a slight hardening before destabilization of the structure at 30.5%.

The [100] test shows the typical behavior for the stress–strain curve, with a drop just after the maximum of stress. This drop expresses in fact a loss of the modified enstatite structure. Normalizing the ITS by the Young’s modulus shows that the different behaviors between the [100] and [010] directions are mostly due to elasticity, since the normalized values, 0.08 and 0.07, are rather similar (Table 5). However, direction [001] stands out with a normalized ISS about twice as large.

**Table 5:** Modified structure

Tensile tests	[100]	[010]	[001]
ITS (GPa)	14.8	7.6	25.6
Corresponding strain (%)	10.5	11.0	23.5
Young’s modulus (GPa)	176	102	186
Normalized stress	0.08	0.07	0.14

Ideal stresses (and associated engineering strains) determined in this study under tensile for the modified enstatite structure. For tensile tests, we report also the Young’s modulus. The normalized stresses are the ideal stresses divided by the elastic modulus

### Ultimate tensile properties of OEN

#### Tension

**Fig. 10:**
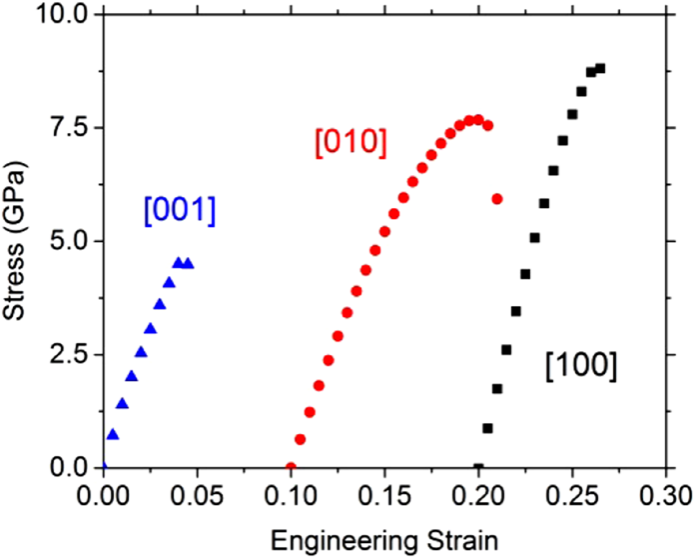
Orthoenstatite. Stress as a function of the engineering strain calculated using a unit cell of enstatite for the tensile test along [100] direction in black square, [010] direction in red circle, and [001] blue triangles

While our tensile test along [010] shows that the mechanical features can be solely attributed to OEN initial structure, we have seen that the tests along [001] and [100] quickly involve another structure. The scope of the study must therefore be restricted to the strains which precede the bond switching responsible for the change in structure to describe the mechanical properties of enstatite. The corresponding stress–strain curves are shown in Fig. 10.

Loading enstatite along [001] leads to the smallest ITS, i.e., 4.5 GPa at a critical strain of 5%. This low value is due to the transition to the modified structure which is more stable under strain. The highest stress is sustained when enstatite is loaded along [100], the instability is reached at 6.0% strain for an ITS of 8.7 GPa, here again due to the transition to the modified structure. In between is the ITS value corresponding to [010] tensile loading: 4.5 GPa at 5.0%.

Between the extreme values of the ITS, we have a ratio of 1.9 showing some anisotropy of orthoenstatite consistent with Young’s modulus. For a more consistent comparison, we computed the normalized ITS (ratio between the ITS and the corresponding Young’s modulus). Normalized ITS are comparable (0.05, 0.07, and 0.04, respectively) for [100], [010], and [001] (Table 6). Although pulling along the largest lattice parameter, i.e., along [100], gives the highest Young’s modulus (Table 6), the different values for the ITS are only due to elastic behavior. Transverse directions are completely relaxed during the tensile tests, so we can determine Poisson ratios (Table 7) according to the transverse lattice parameters’ variations.

**Table 6:** Orthoenstatite

Tensile tests	[100]	[010]	[001]
ITS (GPa)	8.7	7.6	4.5
Corresponding strain (%)	6.0	9.5	5.0
Young’s modulus (GPa)	169	116	122
Normalized stress	0.05	0.07	0.04

Ideal stresses (and associated engineering strains) determined in this study under tensile loading for OEN. For tensile tests, we report also the Young’s modulus. The normalized stresses are the ideal stresses divided by the Young’s modulus

**Table 7:** Orthoenstatite

Tensile tests	[100]	[010]	[001]
*υ*_[100]_	–	0.33	0.14
*υ*_[010]_	0.24	–	0.09
*υ*_[001]_	0.12	0.09	–

Poisson ratio determined in this study under tensile tests for strains below 5%

#### Shear

**Fig. 11:**
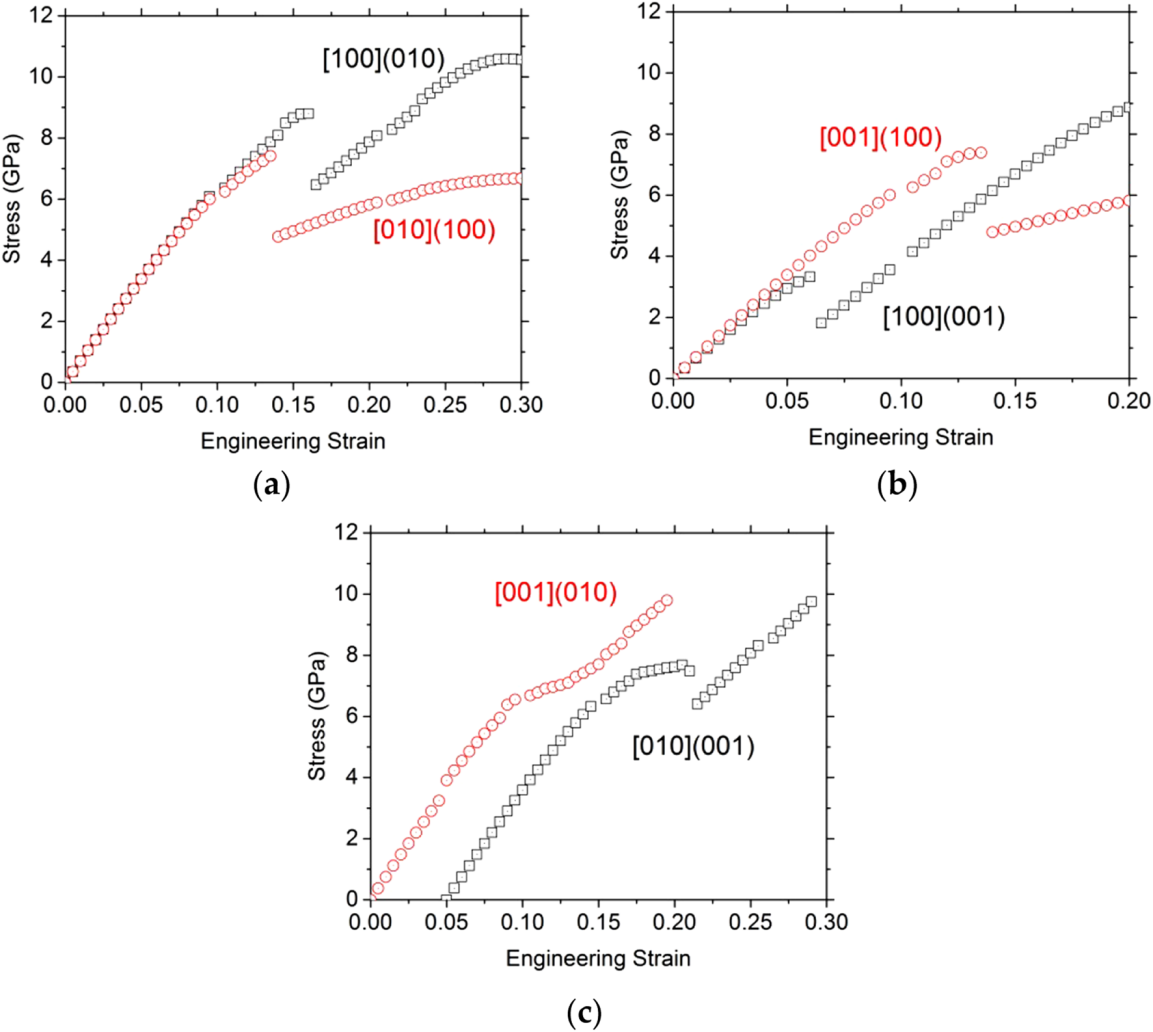
Orthoenstatite. Stress evolution as a function of the engineering strain calculated using a unit cell of enstatite under shear deformation (**a**) for simple shear along [100](010) and [010](100), (**b**) for simple shear along [100](001) and [001](100), and (**c**) for simple shear along [001](010) and [010](001) which is shifted to help the reader

To complete the ideal properties of OEN, we also performed simple shear deformation tests. Six additional simple shear deformation tests have been performed in this study. The evolutions of stress as a function of the engineering strain are shown in Fig. 11.

In contrast to the previous calculations, more specifically the tensile tests along [100] and [001], we have not seen any formation of the modified structure during the shear tests.

The initial slope of the stress strain curves is consistent with the elastic properties of OEN and we observe that the enstatite structure is maintained until a critical strain, associated with generally the maximum stress value. Ideal shear stresses (ISS) range from 7.39 GPa to 8.93 GPa (Table 8). The normalized ISS values (from 0.10 to 0.14) are consistent with ISS value reported for oxides (Ogata et al. 2004) and normalized ISS values obtained for forsterite (Gouriet et al. 2019).

**Table 8:** Orthoenstatite

Simple shear tests	[010](001)	[001](010)	[100](001)	[001](100)	[010](100)	[100](010)
ISS (GPa)	7.48	7.70	8.93	7.39	7.41	8.79
Corresponding strain (%)	16	15.0	20.5	13.5	13.5	16.0
Shear modulus (GPa)	73.3	73.2	66.0	68.5	68.8	68.9
Normalized stress	0.11	0.10	0.14	0.11	0.13	0.12

Ideal stresses (and associated engineering strains) determined in this study under simple shear loading. The normalized stresses are the ideal stresses divided by the shear modulus

**Fig. 12:**
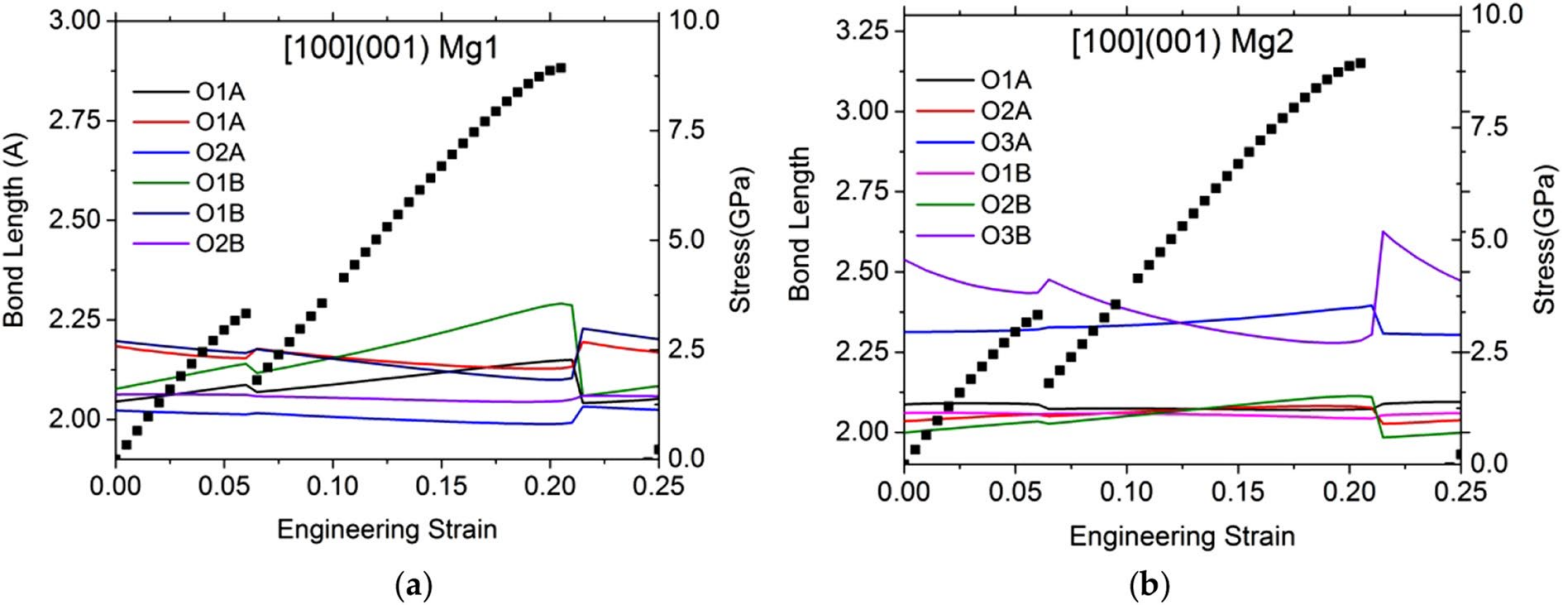
Typical Mg–O-bond length evolution as a function of strain for shear deformation along [100](001) for Mg1 (**a**) and Mg2 (**b**) sites. To help the reader, we plot also the stress–strain curves

Overall, the stability of the structure mainly depends on Mg–O bonds. Indeed, only the stress–strain curve corresponding to the simple shear test along [100](001) shows a small discontinuity at 6% strain (Fig. 11b). However, the Mg–O-bond analysis of this particular shear test does not show a structural modification. The drop on the stress–strain curve at 6% comes from the Mg2–O3B getting closer to each other. Then, for this [100](001) shear test, a major instability occurs at 20.5% strain when the 2 Mg–O bonds are broken (one bond between Mg1 and O1B (Fig. 12a) and the other between Mg2 and O3B (Fig. 12b)). Consequently, the ISS value of [100](001) shear test reaches 8.93 GPa.

For the other shear tests, two behaviors are observed: one with a small hardening (Fig. 11c) behavior before a jump ([001](010) and [010](001)) and one with only an abrupt jump ([001](100), [100](010) and [010](100)) (see Fig. 11a, b). The Mg–O-bond evolutions are showed in Supp. Figure 5 and 6 for [001](100) and [010](001) respectively.

The jumps after 10% of strain on each stress–strain curves (Fig. 11) are due to the breaking of Mg–O bonds: one bond of each Mg in site 1 (Mg1-O1: Suppl. Figure 5a and Suppl. Figure 6a) and two bonds for Mg in site 2 (Mg2–O3A and O3B: Suppl. Figure 5b and Suppl. Figure 6b). When the Mg in site 2 and the both O3 (each in A and B layers) are separated, we observe the O3B exchange with another one. However, at this stage, the OEN is already lost. The hardening behavior appears when the Mg2–O3B becomes shorter and then more stable.

## Concluding remarks

In this work, the main objective is to investigate the mechanical response of OEN to applied strains until it becomes mechanically unstable. This point is reached after the linear elastic regime, which is exhibited by a parabolic regime in all energy–strain curves. In consequences, in all stress–strain curves, a linear regime is observed. This allows us to determine elastic moduli which, with the calculated lattice parameters, validate our calculations.

OEN is quite isotropic under shear loading with ISS value normalized by the shear modulus close to 0.12. The structural analysis shows that the instability occurs when two Mg1–O and Mg2–O bonds are broken, in particular the bond of one Mg with an O3.

During tensile tests, the first stress–strain curve maximums correspond to the ITS of OEN, which are 8.7, 7.6, and 4.5 GPa along the [100], [010], and [001] directions, respectively. The ITS normalized by the Young’s modulus are close to 0.05. During tensile loading along the shortest and the longest lattice parameters, instability appears rapidly during the test (close to 5% of strain). It is important to note that the instability occurs when the system undergoes structural modifications, like broken bonds, leading to structural reconstruction (Jiang and Srinivasan 2013).

Analysis of bond and structure evolution shows that under tensile loading along [100] and [001], OEN gives rise to a modified structure. Under strain, the tetrahedron chain B is stretched, and the O3B–O3B–O3B angle become equivalent to the O3A–O3A–O3A angle in chain A (~ 160°). Consequently, two O3B are exchanged with the Mg in site 2. This oxygen-bond exchange leads to the modified structure. We have characterized some properties of the modified structure by computing the elastic constants, ITS, and the powder X-ray diffraction pattern.

This modified structure becomes more resistant against deformation, with two larger ITS values. Along the [100] direction, the strain reaches 10.5% before the instability occurs, with the ITS value being 1.7 times larger than the ITS value of OEN. Along the [001] direction, the instability is reached at 23.5% of strain, which corresponds to an ITS value 5.7 times larger than the ITS value of OEN. Along the [010] direction, the two structures exhibit the same ITS value with comparable ultimate strains (9.5–11%).

After the case of cementite described by Jiang and Srinivasan (2013), this study shows that in OEN, large strains can lead to Mg–O-bond rearrangements responsible for a significant strain-stiffening. In cementite, this behavior was observed under shear loading. Here, we report the first occurrence of strain-stiffening under tensile loading and we show that this behavior is due to the formation of a modified structure which can be brought at ambient conditions (no applied stress) in a metastable state. Hence, some physical properties of this modified structure are presented.

## Supplementary Information

Below is the link to the electronic supplementary material.

Supplementary file1 (DOCX 1654 KB)

## Data Availability

CIF-files of OEN and the modified structure can be uploaded.
