# Supplementary material for: Ultimate mechanical properties of enstatite
Source: Phys Chem Miner. 2022 Jul 11;49(8):30. doi: 10.1007/s00269-022-01206-5 (PMC9276559; doi:10.1007/s00269-022-01206-5)
Supplement: Supplementary file 1 — Supplementary file1 (DOCX 1654 KB) [file 269_2022_1206_MOESM1_ESM.docx]

Supplementary materials:

| 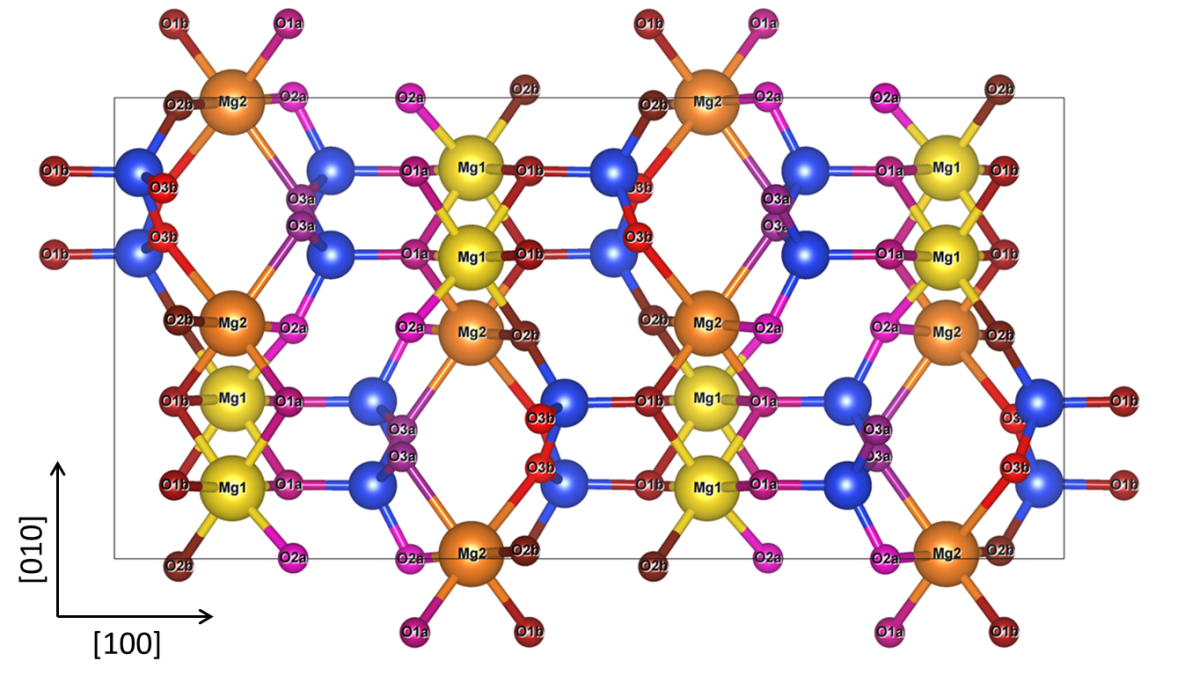(a) |
| --- |
| 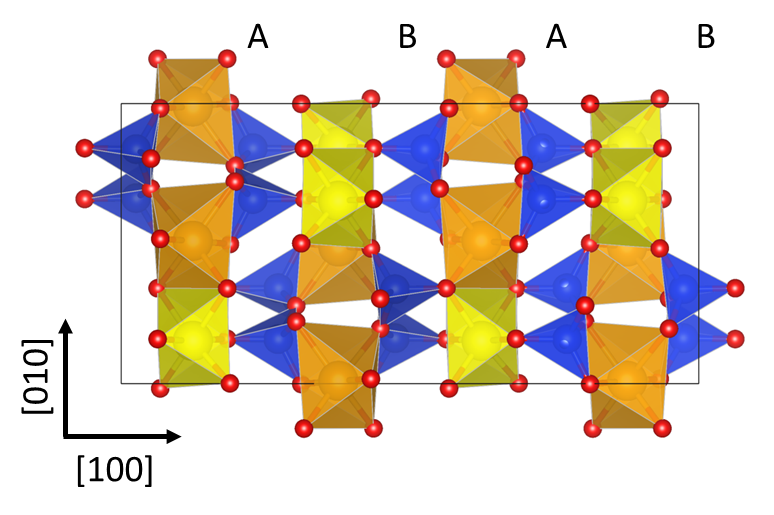(b) |

**Supp. Figure 1.** Orthoenstatite structure: (**a**) Ball-and-stick representation. We applied different colors for each site of Mg and O for the *Pbca* relaxed enstatite structure: Mg1 in yellow, Mg2 is in orange, O in layer A are in a shading magenta and O in layer B in shading of red. (**b**) Polyhedral representation. Mg1 is in yellow, Mg2 is in orange, O is in red.

| 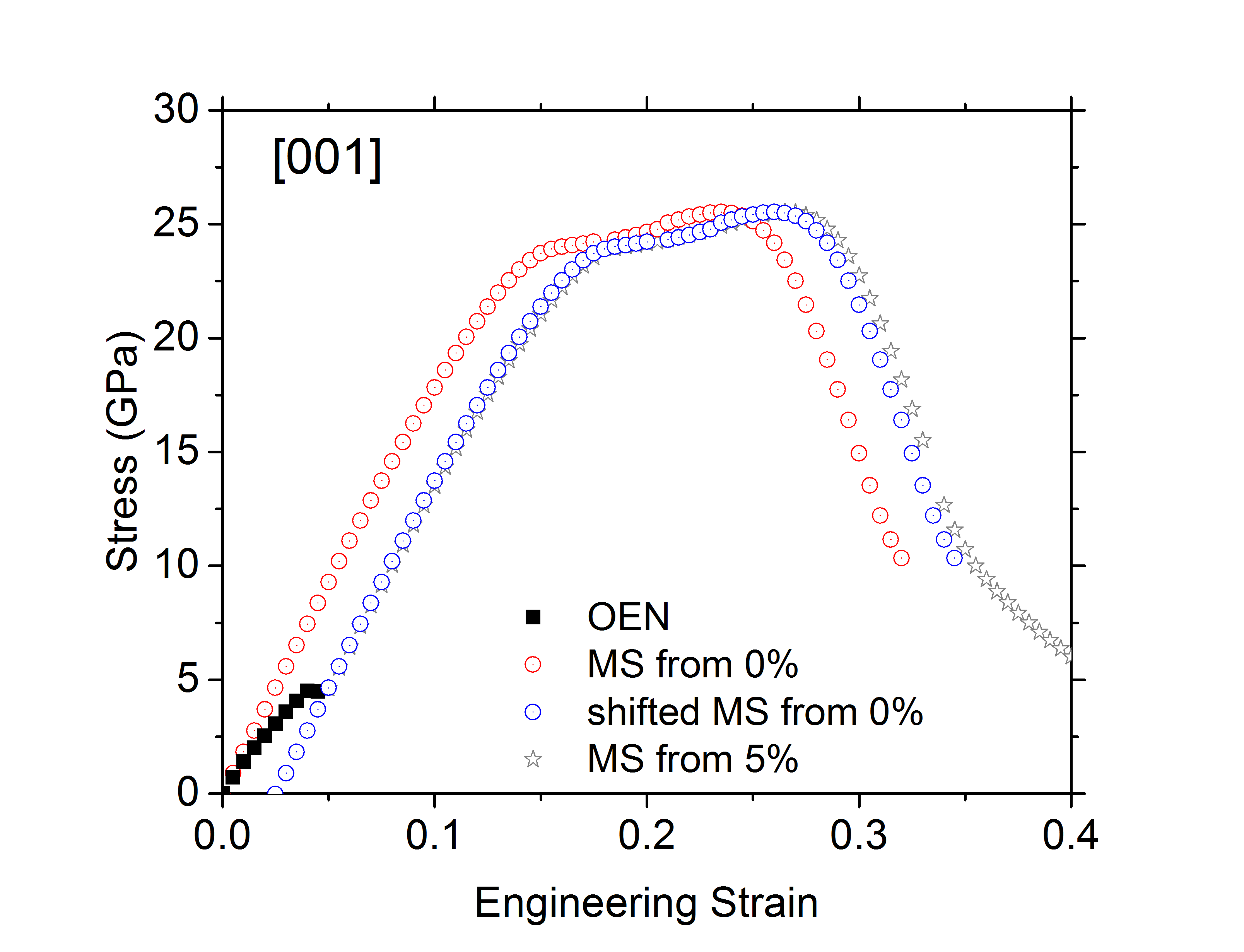 | 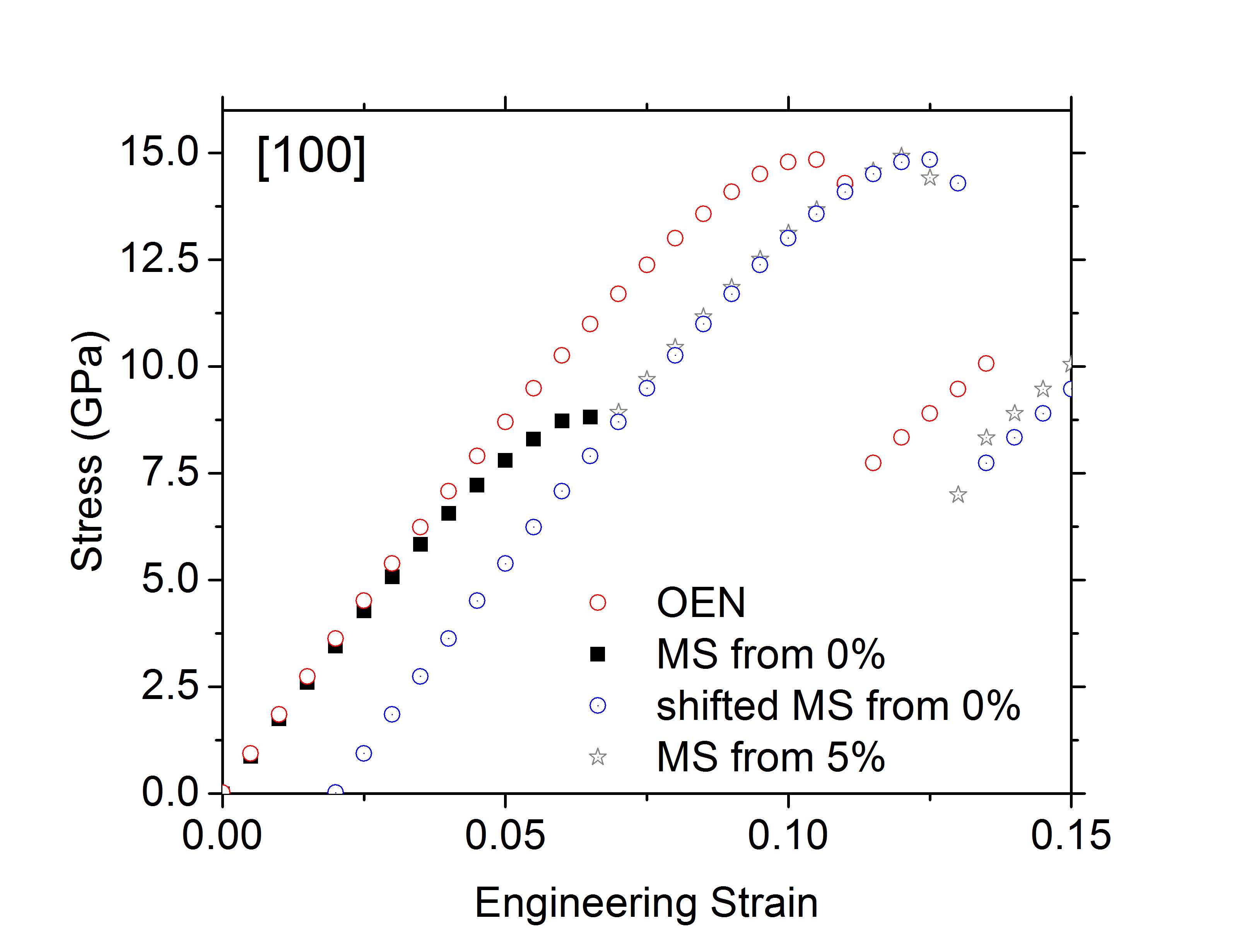 |
| --- | --- |
| (a) | (b) |

**Supp. Figure 2.** Evolution of stress as a function of the engineering strain along the (**a**) [001] and (**b**) [100] direction for the Orthoenstatite (black square), the modified structure (red circle). The stress-strain curve of the new structure is shifted by 0.025/0.02 (blue empty circle). The stars correspond to the results from the test with OEN as starting point.

| 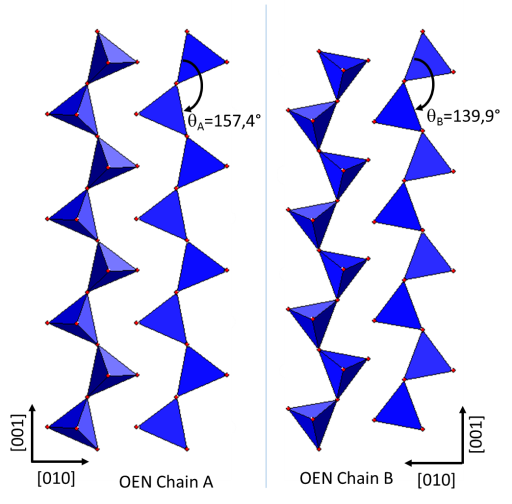 | 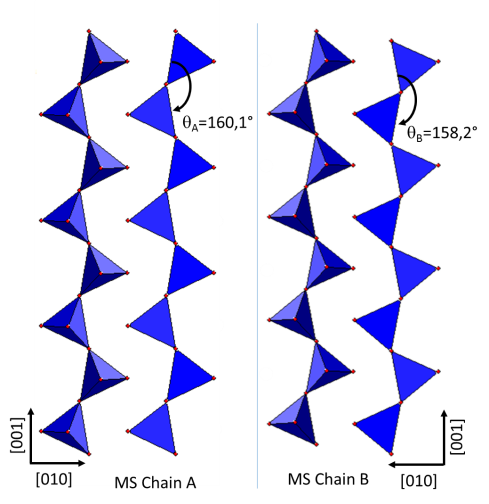 |
| --- | --- |
| (a) | (b) |

**Supp. Figure 3.** Tetrahedron chains A and B for (**a**) the orthoenstatite (OEN) and (**b**) the modified structure (MS). The O3-O3-O3 angles for both chains (θ_A_ and θ_B_) are shown.

| 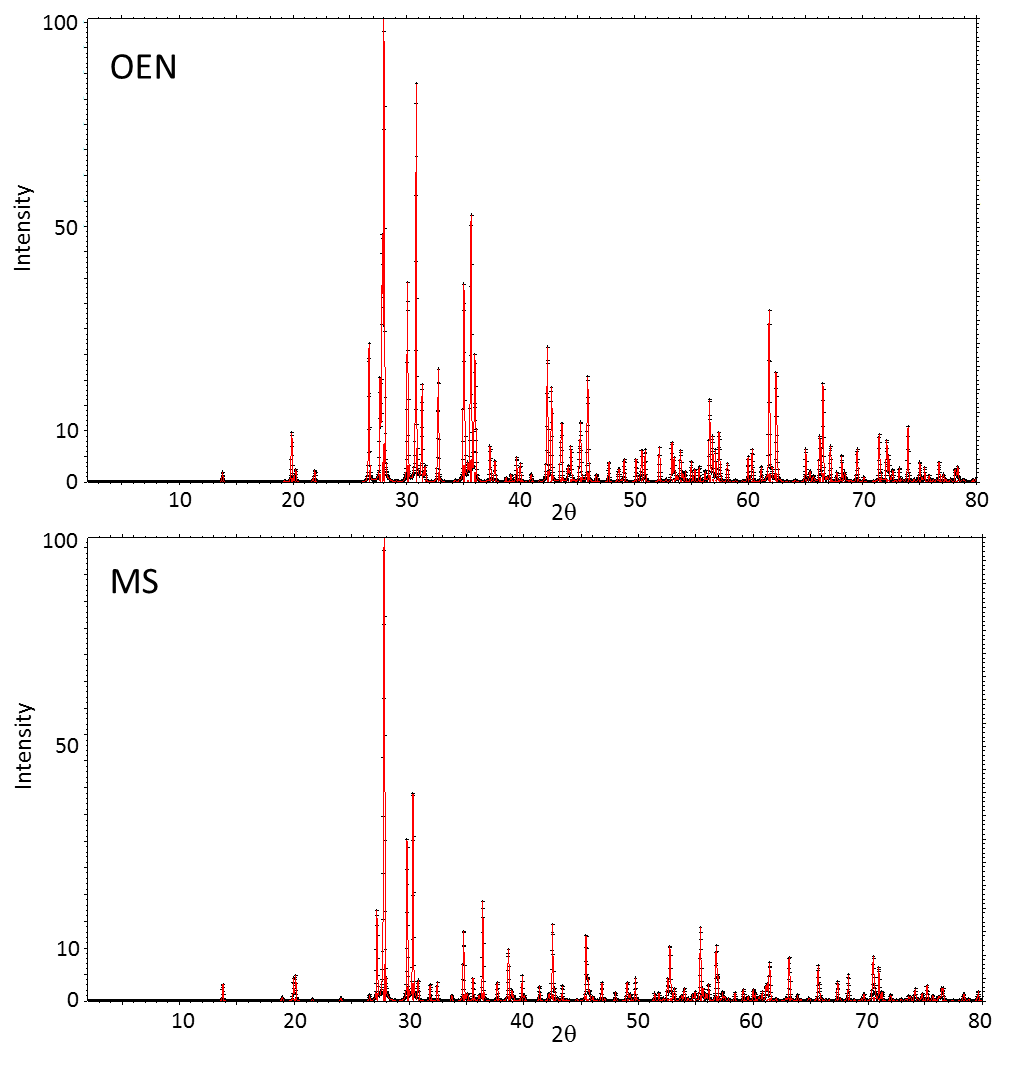 |
| --- |

**Supp. Figure 4.** Calculated powder X-ray diffraction using Cu *K*α_1_ radiation for the orthoenstatite (OEN) and the modified structure (MS).

| 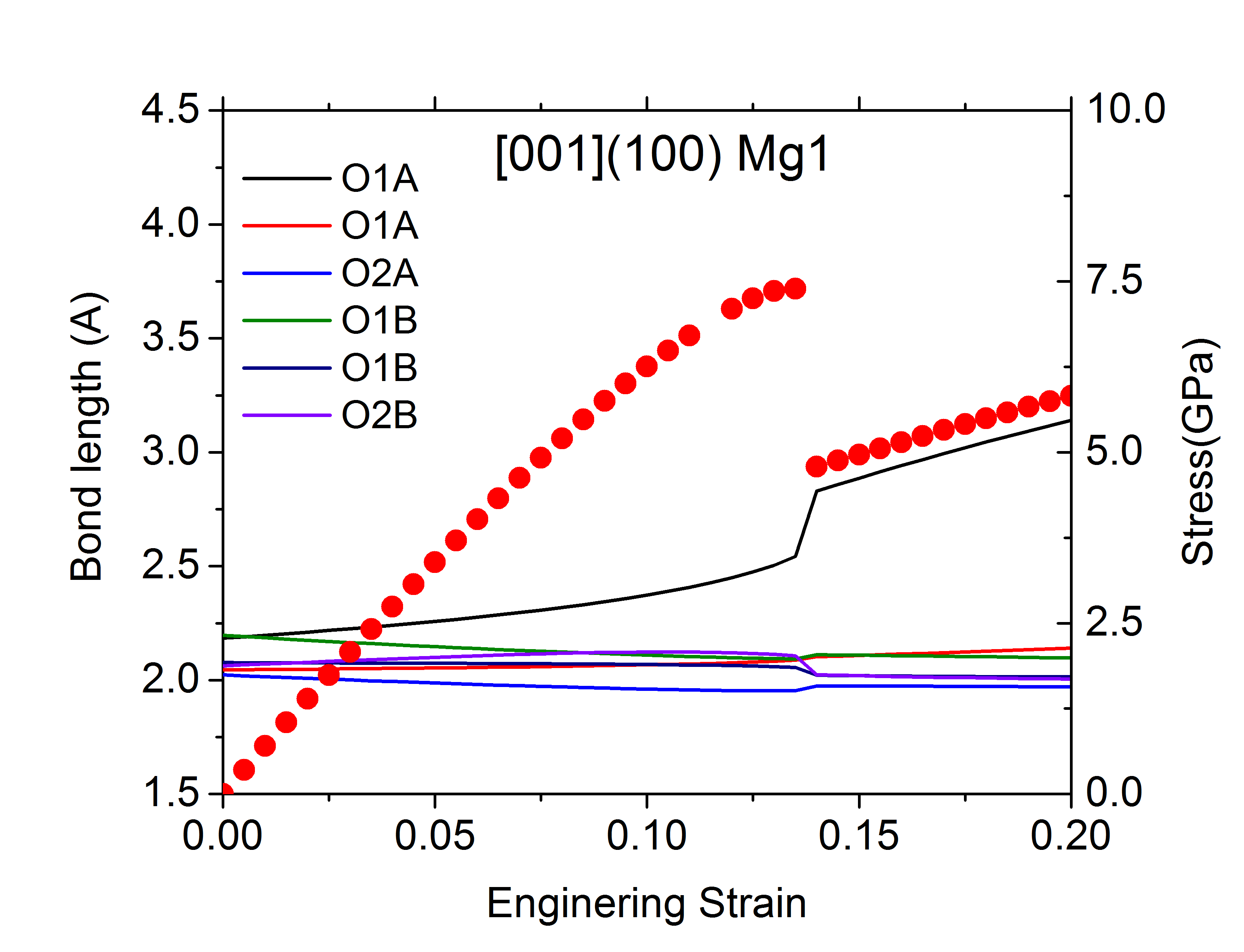 | 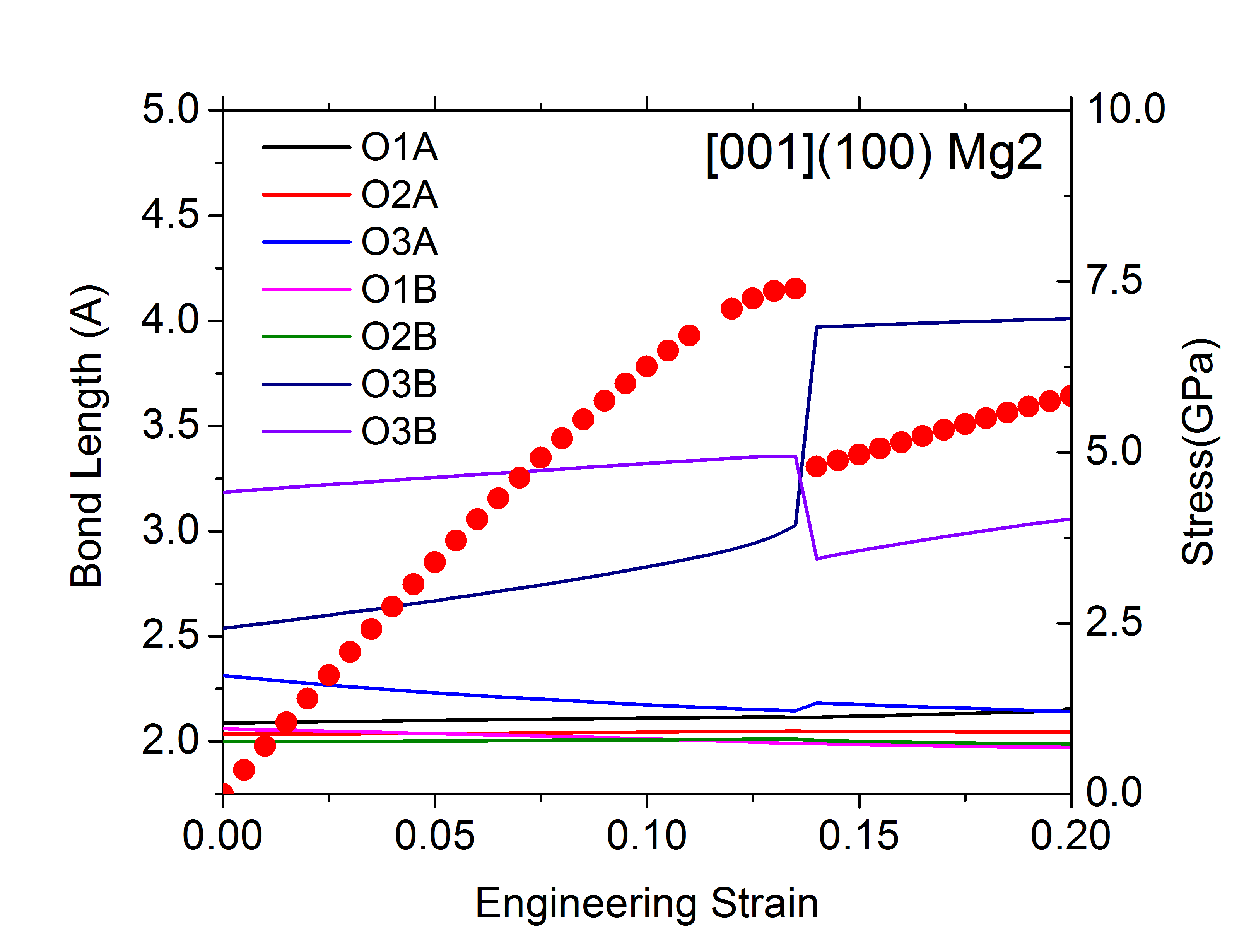 |
| --- | --- |
| (**a**) | (**b**) |

**Supp. Figure 5.** Typical Mg-O bond lengths evolution as a function of shear strain along [001](100) for Mg on the same chain (**a**) the Mg in site 1, (**b**) the Mg in site 2. To help the reader, we plot also the stress-strain curves.

| 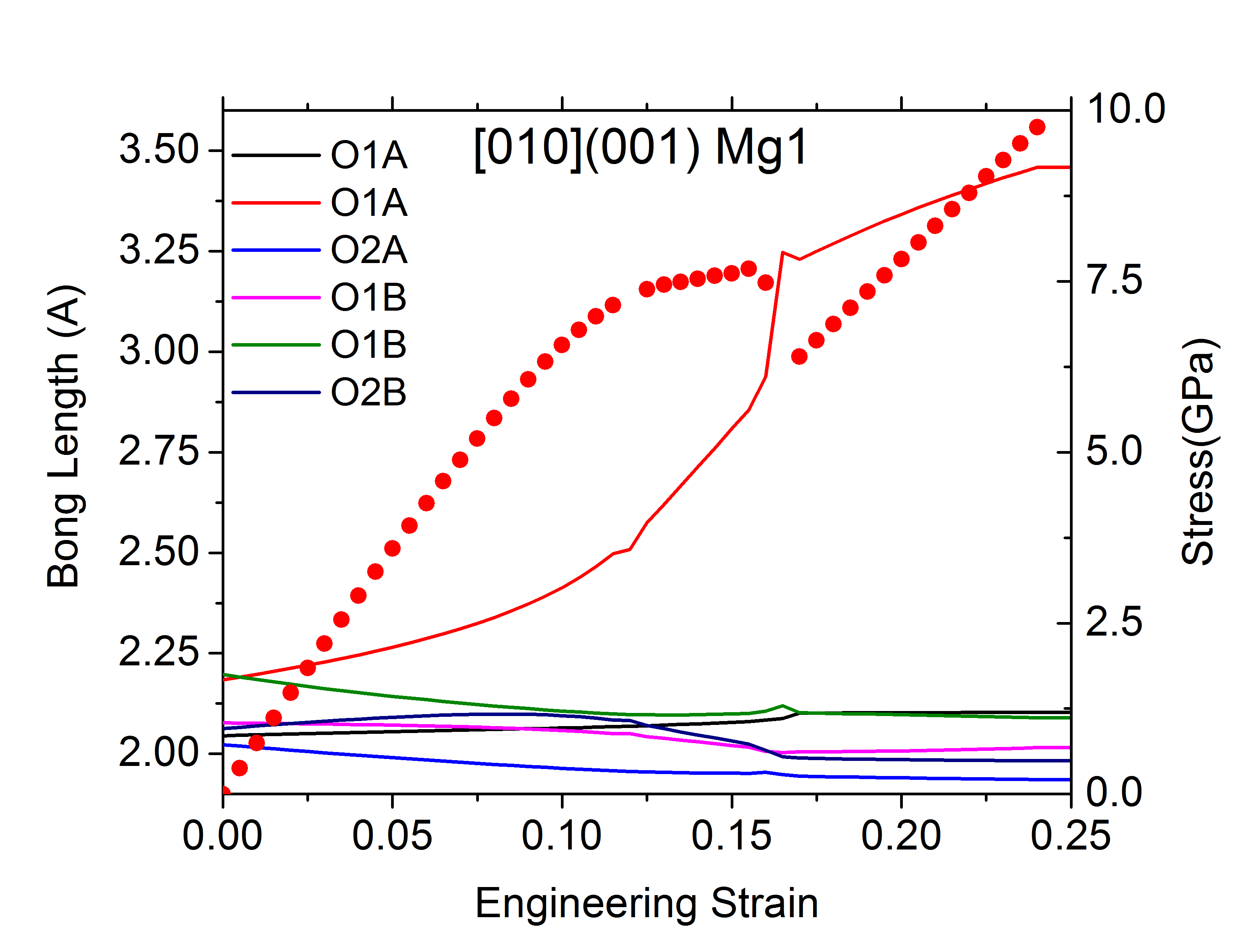 | 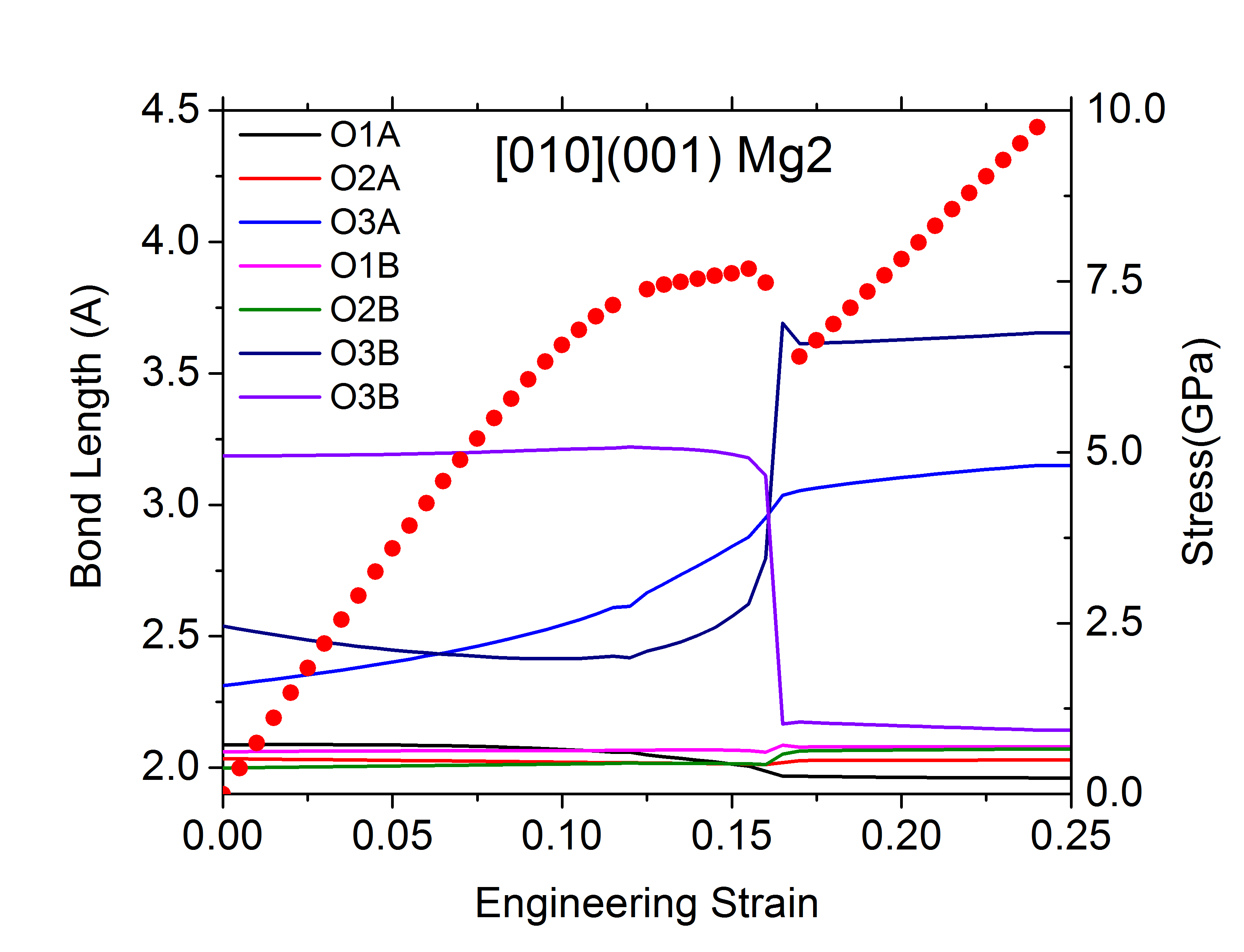 |
| --- | --- |
| (**a**) | (**b**) |

**Supp. Figure 6.** Typical Mg-O bond lengths evolution as a function of shear strain along [010](001) for Mg on the same chain (**a**) the Mg in site 1, (**b**) the Mg in site 2. To help the reader, we plot also the stress-strain curves.
